# 5-Azacytidine inhaled dry powder formulation profoundly improves pharmacokinetics and efficacy for lung cancer therapy through genome reprogramming

**DOI:** 10.1038/s41416-020-0765-2

**Published:** 2020-02-27

**Authors:** Philip J. Kuehl, Carmen S. Tellez, Marcie J. Grimes, Thomas H. March, Mathewos Tessema, David A. Revelli, Larry M. Mallis, Wendy W. Dye, Tyler Sniegowski, Aaron Badenoch, Michael Burke, Devon Dubose, David T. Vodak, Maria A. Picchi, Steven A. Belinsky

**Affiliations:** 1 Lung Cancer Program, Lovelace Respiratory Research Institute, Albuquerque, NM USA; 2 Bend Research Inc., Bend, OR USA

**Keywords:** Non-small-cell lung cancer, Cancer therapy

## Abstract

**Background:**

Epigenetic therapy through demethylation of 5-methylcytosine has been largely ineffective in treating lung cancer, most likely due to poor tissue distribution with oral or subcutaneous delivery of drugs such as 5-azacytidine (5AZA). An inhalable, stable dry powder formulation of 5AZA was developed.

**Methods:**

Pharmacokinetics of inhaled dry powder and aqueous formulations of 5AZA were compared to an injected formulation. Efficacy studies and effect of therapy on the epigenome were conducted in an orthotopic rat lung cancer model for inhaled formulations.

**Results:**

Inhaled dry powder 5AZA showed superior pharmacokinetic properties in lung, liver, brain and blood compared to the injected formulation and for all tissues except lung compared to an inhaled aqueous formulation. Only dry powder 5AZA was detected in brain (~4-h half-life). Inhaled dry powder was superior to inhaled aqueous 5AZA in reducing tumour burden 70–95%. Superiority of inhaled 5AZA dry powder was linked to effectively reprogramming the cancer genome through demethylation and gene expression changes in cancer signalling and immune pathways.

**Conclusions:**

These findings could lead to widespread use of this drug as the first inhaled dry powder therapeutic for treating local and metastatic lung cancer, for adjuvant therapy, and in combination with immunotherapy to improve patient survival.

## Background

Novel targeted- and chemo-therapies for lung cancer (LC) have achieved modest improvement in median survival for advanced LC, but offer no clear path to treatments that could make this a chronic, rather than fatal disease.^1^ Furthermore, for the majority of the more than 220,000 new LC cases diagnosed annually in the US for which most targeted therapy is not an option, durable responses with chemotherapy are uncommon and median survival after relapse is 6.5 months.^2^ A major recent advance in LC is the integration of immune checkpoint inhibitors with chemotherapy; however, only 34% of Stage IV patients with good ECOG performance status of 0 or 1 remained alive and progression free at 12 months post therapy initiation.^3–5^

The Cancer Genome Atlas (TCGA) has interrogated over 800 non-small cell lung cancers (NSCLCs) and revealed that most tumours contain hundreds of genes with cytosine methylated promoter regions associated with reduced transcription.^6,7^ Epigenetic therapy, through its ability to activate these genes, offers a strategy that could produce durable and sustained tumour regression. Cytosine methylation is dominant in transcriptional repression, and inhibitors of the cytosine DNA-methyltransferases, 5-azacytidine (5AZA) and 5-aza-2’-deoxyazacytidine (DAC), can induce re-expression of genes silenced through promoter hypermethylation.^8,9^ These drugs, delivered at doses much lower than the maximum tolerated dose, are serving as potent therapy for myelodysplasia with an overall response rate (ORR) of >60%, leading to FDA approval for treatment of these diseases.^10,11^ Importantly, while histone deacetylation inhibitors (HDACi) are not very effective in inducing re-expression of genes silenced by methylation, such inhibitors can synergise with demethylating agents to relieve transcriptional repression.^12^ This combination therapy was used in a Phase 1/2 trial in which LC patients who progressed on other therapies were treated with 5AZA and the HDACi entinostat. This therapy was well tolerated, 10 of 34 evaluable patients had stable disease (29%) with one partial, and one complete response.^13^ The lack of replication of findings from this trial may stem from poor distribution of 5AZA to the lungs.

5AZA and DAC are unstable in an aqueous solution being subject to hydrolysis and are substrates for catabolism by cytidine deaminase.^14,15^ While intravenous (IV) or subcutaneous administration of 5AZA will avoid hepatic first pass, sequence variants within the promoter region of cytidine deaminase have been associated with variation in enzymatic activity leading to reduced half-life of 5AZA and poorer outcome for patients with myelodysplasia.^14–16^ Pharmacokinetic studies in mice show that IV administration of 5AZA resulted in high concentrations in peripheral blood, but only trace amounts were detected in lung.^17^ Oral formulations of 5AZA are subject to hepatic first pass and toxicity in the gastrointestinal tract limits the deliverable dose.^18,19^ These barriers could be mitigated by inhaled delivery of 5AZA where direct deposition and absorption into the bronchial and pulmonary blood supply, which avoids hepatic first pass should achieve therapeutic drug concentrations in local tumours with subsequent systemic delivery to potentially treat metastases.^20,21^

We have developed an orthotopic LC model in which xenografts of human LC-derived cell lines are efficiently engrafted throughout the lungs of the Rowett nude rat.^22^ Our first study demonstrated that combination therapy, with systemic delivery of 5AZA and entinostat at doses and schedule similar to the Phase 2 clinical trial, were synergistic in suppressing tumour growth and induced reprogramming of the epigenome as detected by gene demethylation and re-expression.^23^ We extended this work through developing a highly respirable aqueous aerosol formulation of 5AZA delivered via a nebuliser. The inhaled, nebulised delivered lung dose of 0.6 mg/kg compared to a systemic dose of 2 mg/kg 5AZA yielded an improved pharmacokinetic profile in the lung, equivalent reduction in tumour burden, and enhanced commonality for demethylation of 300 genes in tumours sampled throughout lung lobes at one-third of the effective systemic dose.^24^ Qiu et al. validated our findings in an orthotopic mouse model.^25^

Nebulising 5AZA for therapy is constrained by the ~90 min needed to deliver the required dose in a clinical setting and a poor systemic pharmacokinetic profile that would limit effectiveness for treating metastatic disease present in ~60% of lung cancer patients at diagnosis.^2^ Dry powder drug formulations are generally more stable and can be delivered via a breath-actuated, hand-held single dose inhaler in a matter of minutes. Thus, the goals for this study were to develop a stable inhalable dry powder formulation of 5AZA, characterise its PK properties for local and systemic delivery, and conduct therapeutic efficacy studies using our orthotopic nude rat lung cancer model in conjunction with evaluating reprogramming of the epigenome and transcriptome.

## Methods

### Manufacturing and in vitro characterisation of spray-dried powder of 5AZA

5AZA (Toronto Research Chemicals, Canada) was dissolved at 5 mg/ml in DMSO (10% solvent mass) and H_2_O. The excipients trehalose and L-leucine at a ratio of 80/20 in H_2_O were added for stability and particle formation just prior to atomisation. The solution was spray-dried using a two-fluid atomisation nozzle with a small-scale custom spray dryer (BLD-35). Details describing the characterisation of the aerosol are provided in Supplementary Methods.

### Treatment protocols and tumour cell implantation

Male Sprague Dawley rats, 6-8-weeks-old were obtained from Charles River CD. For pharmacokinetic studies, rats (3/group/time point) were treated with a single dose of 5AZA dissolved in saline administered systemically by intraperitoneal injection (2 mg/kg), as an aerosol dissolved in H_2_O (0.6 mg/kg inhaled deposited dose) with three Pari LC Plus nebulisers and delivered to the rats by nose-only exposure, or as an inhaled dry powder (0.3, 0.6, 0.9 mg/kg inhaled deposited dose) generated by a rotating brush generator for nose-only delivery as described in detail in Supplementary Methods.^26^ The aerosol dose delivered to the rat lungs was calculated based on the aerosol concentration times the respiratory minute volume times the exposure period times the deposition fraction divided by body weight.^26–28^ Animals were serially sacrificed for collection of plasma, lung, liver and brain tissue at 11 (systemic dose) or 10 (aerosol exposure) time points over 12 h post exposure. At euthanasia, plasma, lung, liver and brain were collected and stored at −80 °C until analysis. Blood samples (≤4 mL) were collected into K_3_EDTA tubes containing 80 µL of a 12.5 µg/mL solution of the cytidine deaminase inhibitor tetrahydrouridine (THU) to prevent degradation of 5AZA prior to analysis.^29^ Tissues were also collected in THU (lung 30 µL, brain 40 µL, and liver 155 µL; 12.5 µg/mL solution) prior to freezing.

Male Rowett nude rats (Cr:NIH-ru), 4–6-weeks-old were obtained from Envigo (Indianapolis, IN). Calu6, Calu3, and H358 cells obtained from American Type Culture Collection (Manassas, VA) and RH2 cells obtained from Steven Dubinett were cultured and instilled via orotracheal intubation as described.^22,23^ Cell line authentication was performed by Genetica DNA laboratories within the last year. Rats (15/group) received Calu6, Calu3 (15 million cells/rat), or H358 (7.5 million cells/rat due to greater efficiency for engraftment) tumour cells via intratracheal instillation. Pilot studies with RH2 cells also showed greater engraftment and very aggressive growth, thus rats received 7.5 million cells and the sample size was increased to *n* = 20/group to ensure comparable sample sizes for the treatment period. Three weeks following instillation of tumour cells, rats were exposed to air (untreated), inhaled aqueous or dry powder 5-AZA (0.6 mg/kg lung dose) for 4 consecutive days over 4 consecutive weeks as described.^23,24^ An additional six rats that did not receive tumour cells were included to determine tumour-free lung weights for comparison with treatment groups.

### Bioanalysis of 5AZA

Plasma and homogenised tissues were analysed via a new LC-MS/MS as described in detail under Supplementary Methods.

### Tissue collection and estimation of tumour burden

Prior to sacrifice, five animals from each treatment group and air were randomly selected for collection of tumours (*n* = 12 per animal) for molecular assays. Animals were euthanised by intraperitoneal injection of an overdose of a barbiturate-based sedative. Due to the near curative effect of treatment for H358 tumours, only four tumours each were identified and/or large enough (≥2 mm) to support assays from the treatment groups. Lungs were weighed and then inflated with 10% neutral-buffered formalin. Paraffin embedded lungs were sectioned at 5 μm thickness and stained with haematoxylin and eosin. Our previous study demonstrated that treatment-related reduction in tumour burden was highly correlated with estimates of tumour volume.^23^ Therefore, tumour burden was used to assess the response to systemic versus aerosol delivery of 5AZA. Tumour burden was quantitated as the change in normal lung weight (average of the naïve rat lungs) compared with tumour bearing lung weights in the untreated and 5AZA treatment groups.

### Gene methylation profiling

DNA was isolated from four untreated tumours (1 from each rat), 10 tumours each from dry powder- and aqueous-exposed rats (either three or two tumours per lung to equal a total of 10) for Calu6, Calu3, and RH2 as described.^23^ Due to the near curative effect of treatment for H358 only four discernible tumours from each treatment were available to support methylation and expression studies below. DNA from normal lung tissue obtained from five, cancer-free smokers was included to identify genes methylated in normal cells. Bisulfite-modified DNA was hybridised to Illumina Infinium Methylation EPIC Beadchips (Illumina, San Diego, CA).

### Gene transcriptome profiling

Total RNA was extracted from the same untreated and treated tumours studied for methylation following TRI-reagent (Sigma, St. Louis, MO) instructions and quantified using a NanoDrop 2000 spectrophotometer and Qubit 2.0 Fluorometer (Life Technologies). RNA integrity was assessed with an Agilent 2100 Bioanalyzer and an RNA Integrity Number (RIN) extracted from the electropherogram to determine the RNA quality. RNA libraries with PolyA selection were prepared and sequenced at 150 bp paired-end runs at a target depth of 30 million reads per sample using an Illumina HiSeq (Genewiz, San Diego, CA).

### Statistical analyses

Pharmacokinetic parameters were estimated for plasma, lung, liver and brain using Phoenix WinNonlin version 6.2 software (Certara L.P.) using a non-compartmental approach. Details are provided under Supplementary Methods. The two-sample *t*-test and analysis of variance were used to compare tumour burden between the two treatment groups and the two groups with the air, respectively.

Due to the strong association between methylation of CpGs around the transcriptional start site (TSS) and gene silencing, our analytic strategy for methylation data focused on this region to assess the methylation status of 179,314 CpG oligonucleotide probes within 200 base pairs 5’ of the TSS and extending through the first exon. Details are provided in Supplementary Methods. Briefly, average signal intensity between methylated and unmethylated probes was determined, and β-values from 0-1 (fully methylated) were calculated. Genes whose average β-values were ≥0.2 across the interrogated region in normal lung tissue were excluded from further analysis. Average β-values ≥0.45 across CpGs within gene promoters were scored as positive for methylation in untreated tumours and a reduction in β-value of ≥30% for a methylated gene in treated tumours was scored as demethylation. Analyses were conducted with SAS 9.4.

RNA sequencing data were analysed using Illumina’s cloud-based genomics-computing environment as described in detail under Supplementary Methods. Heatmaps were generated in R. The colour gradation reflects the z-score ranking of rlog values from DESeq2 analyses.

Genes from pathways affected in cancer were compiled from Biocarta, mSigdb and IPA knowledge base and used to assess over-enrichment in significantly differentially expressed genes (FDR < 0.01) in each group of treated tumours. Qiagen Ingenuity Pathway Analysis software was used to identify pathways statistically over-represented in the lists of differentially expressed genes.

## Results

### Development and characterisation of a dry powder formulation of 5AZA

The key to development of an inhalable formulation of 5AZA is maintaining its stability during the spray drying process, since 5AZA rapidly degrades in aqueous solution at room temperature. To achieve this, several diluents were evaluated that included ammonium acetate buffer, unbuffered water, 1:1 ethanol: water, and DMSO. DMSO (10%) provided the best chemical stability and solubility and was mixed with 5AZA to prevent degradation during the spray drying process. The standard excipients trehalose and leucine were then solubilised in water and in-line mixed with the DMSO-5AZA just prior to atomisation. The composition of the final formulation after spray drying was 70/20/10 (w/w) trehalose/leucine/5AZA with 10% residual DMSO. Particle size distribution indicated a homogeneous particle size distribution that was <10 µm with 50% of the particles <3.3 µm. Scanning electron microscopy revealed that the formulation was composed of primary particles with no signs of fusion or agglomeration. HPLC analysis revealed purity greater than 92% with no degradation peaks detected after spray drying or following vacuum desiccation (approach used for stable storage). The particle size distribution was measured with a next generation impactor and a clinical device (RS01 low resistance, Plastiape) at 4kPA. The mass median aerodynamic diameter (MMAD) and geometric standard deviation (GSD) was 3.58 ± 1.58 µm, a value that meets the requirements by the FDA for an orally inhaled aerosol.

### Superior pharmacokinetic profile for the dry powder formulation of 5AZA

**Fig. 1:**
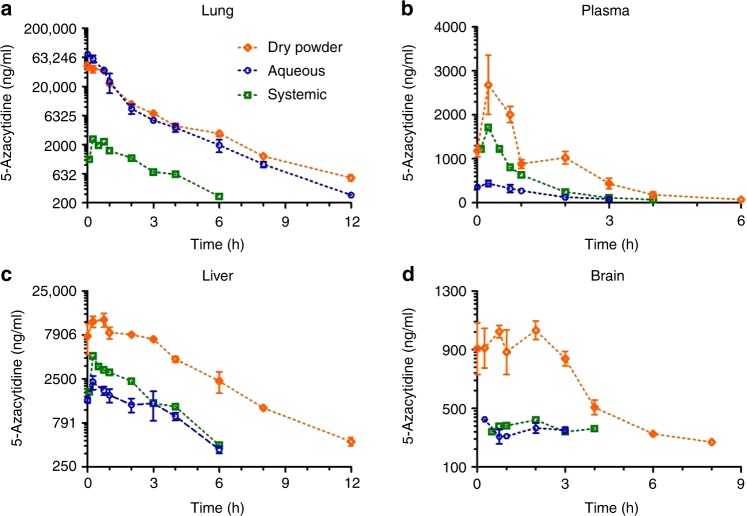
Superior pharmacokinetic profile for inhaled dry powder 5AZA. Rats were exposed to a single dose of 5AZA (inhaled dry powder [0.6 mg/kg lung dose], inhaled aqueous [0.6 mg/kg lung dose], or systemic (2 mg/kg, i.p.) and sacrificed at multiple time points over 12 h post exposure to compare pharmacokinetic profiles in plasma (**a**), lung (**b**), liver (**c**) and brain (**d**) as defined by C_max_, AUC, and half-life (Table 1). *n* = 3/time point; mean ± SD.

5AZA (2 mg/kg) was administrated by intraperitoneal injection to compare pharmacokinetics to our previously studied inhaled aqueous formulation (0.6 mg/kg lung dose) versus the inhaled dry powder formulation (0.3, 0.6, 0.9 mg/kg lung doses). The pulmonary deposited doses were all calculated based on average group body weights, actual measured 5AZA aerosol concentrations at the breathing zone of the nose only inhalation exposure system via standard inhalation drug delivery methods.^26,28,31^ The 5AZA solution nebuliser aerosols consistently resulted in particle size distributions of ~1.5 μm MMAD (GSD ~1.6) and the inhalation dry powder 5AZA consistently resulted in particle size distributions of ~3 μm MMAD (GSD ~1.7). The equivalent deposited doses of the inhaled dry powder and aqueous 5AZA (0.6 mg/kg dose) showed comparable pharmacokinetic profiles in the lung that were greatly superior to systemic delivery with respect to the peak drug concentration C_max_ (~30-fold) and AUC (15-fold [Table 1, Fig. 1a]). In addition, a significant dose-dependent increase in AUC was seen with the dry powder formulation (Table 1). There was a 2.5- and 6-fold increase in AUC and a 1.5- and 6-fold increase in C_max_ for the inhaled dry powder (0.6 mg/kg) compared to systemic (2 mg/kg) and inhaled aqueous (0.6 mg/kg) 5AZA (Table 1, Fig. 1b). A dose-dependent increase in plasma AUC was seen with the dry powder (Table 1). The pharmacokinetic analyses in the liver also demonstrate the superiority of the dry powder 5AZA compared to the systemic dosing (AUC increased 4-fold; C_max_ increased 2.5-fold) and the aqueous formulation (AUC increased 6.5-fold; C_max_ increased 5-fold; [Table 1, Fig. 1c]). The oral bioavailability of 5AZA is low and unlikely to contribute to the systemic profile.^32^

**Table 1:** Comparison of pharmacokinetic profile for 5AZA delivered systemically versus inhalation of aqueous or dry powder formulations in plasma and tissues.

		Lung			Plasma		
Delivery route	Dose (mg/kg)	AUC (h*ng/ml)	Cmax (ng/ml)	Half-life (min)	AUC (h*ng/ml)	Cmax (ng/ml)	Half-life (min)
Systemic	2.0	5740 ± 179	2500 ± 43	116	1,810 ± 115	1715 ± 135	54
Aerosol-Aq	0.6	87,400 ± 6800^a^	71,300 ± 5490	125	678 ± 54^a^	442 ± 67	69
Aerosol-DP	0.3	38,600 ± 2030	24,500 ± 2380	124	2430 ± 187	1740 ± 269	51
Aerosol-DP	0.6	82,900 ± 3120^a,c^	44,900 ± 7040	143	4270 ± 343^a,b,c^	2680 ± 673	70
Aerosol-DP	0.9	144,000 ± 10,180^c^	99,700 ± 11,000	161	7040 ± 564^c^	3220 ± 644	68

		Liver			Brain		
Delivery route	Dose (mg/kg)	AUC (h*ng/ml)	Cmax (ng/ml)	Half-life (min)	AUC (h*ng/ml)	Cmax (ng/ml)	Half-life (min)
Systemic	2.0	10,600 ± 429	4560 ± 245	114	1400 ± 90	421 ± 86	NC
Aerosol-Aq	0.6	7150 ± 609^a^	2300 ± 405	102	1010 ± 42	425 ± 0	NC
Aerosol-DP	0.3	12,400 ± 623	5000 ± 689	130	1430 ± 125	610 ± 185	NC
Aerosol-DP	0.6	45,400 ± 2000^a,b,c^	11,800 ± 2090	154	4940 ± 157^a,b,c^	1030 ± 64	263
Aerosol-DP	0.9	32,600 ± 1130^c^	11,200 ± 1550	110	7340 ± 319^c^	1630 ± 259	177

*Aq* aqueous, *DP* dry powder.

^a^*p* < 0.01 when using the linear model to examine the difference between 5-AZA delivery routes comparing aerosol dry powder (0.6 mg/kg) or aerosol aqueous (0.6 mg/kg) to systemic (2 mg/kg).

^b^Indicates significant increase compared to both systemic and inhaled aqueous.

^c^*p* < 0.01 when using the linear model to examine the difference between 5-AZA aerosol dry powder doses using 0.3 mg/kg as the reference.

Metastasis to the brain remains a daunting challenge for therapy due in large part to drug delivery. Surprisingly, there were dramatic differences in brain PK for 5AZA by drug composition and exposure route. A low level of 5AZA approaching limits of quantitation was observed with systemic dosing and inhaled delivery of the aqueous formulation (Fig. 1d). In contrast, inhalation of the 0.6 mg/kg dry powder 5AZA resulted in a C_max_ and AUC of 1032 ng/ml and 4940 h*ng/ml with a drug half-life of ~4 h (Table 1, Fig. 1d). The brain to plasma AUC ratio was 1.5 and 1.15 for the aqueous and inhaled dry powder 5AZA formulations. However, at the same pulmonary deposited dose the exposure to the brain was significantly increased for the inhaled dry powder 5AZA.

### Improved therapeutic efficacy for the dry powder formulation of 5AZA

**Fig. 2:**
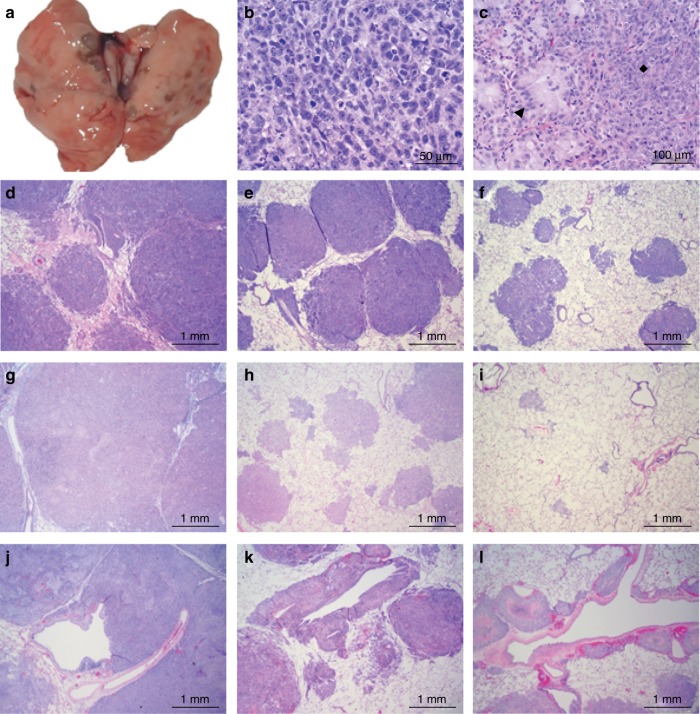
5AZA treatment effects the pathology of orthotopically xenografted human lung tumours. Gross (**a**) and microscopic images (**b**–**l**) of human carcinoma orthotopic xenografts in the lungs of nude rats. At 2.5 weeks post instillation, RH2 squamous cell carcinoma cells established extensive pinkish-tan nodules that irregularly elevated more than two-thirds of the pleural surface (**a**). Carcinomas from Calu6 cells (**b**) contained anisokaryotic, pleomorphic cells arranged in solid expanses and nests among a network of fine fibrovascular stroma. Nuclei were unoriented and irregularly shaped. Numerous mitotic figures and scattered nuclear debris were present. The Calu3 xenografts (**c**) were dimorphic with populations of cells that had differentiated features of an adenocarcinoma (arrowhead) juxtaposed to solid expanses of pleomorphic cells (diamond). All lungs instilled with Calu6 cells (**d, e, f**), H358 cells (**g, h, i**) and RH2 cells (**j, k, l**) contained carcinomas, but in lungs from untreated controls (**d, g, j**), parenchyma and peripheral bronchiolovascular tracts were nearly completely obliterated in extensive portions of the sections by large coalesced nodules of tumour tissue. In lungs from animals treated with aqueous (**e, h, k**) and dry powder 5AZA (**f, i, l**), less of the section was occupied by tumour tissue. Peribronchiolar and perivascular expanses were less extensive, and small nodules were often more isolated within the parenchyma. The H358 nodules were particularly small and sparse in lungs from animals treated with the dry powder 5AZA formulation (**i**). Necrosis was extensive in RH2 xenografts in 5AZA-treated animals’ lungs (bright eosinophilic foci, often centralised, within tumours in **k, l**).

An efficacy study comparing inhaled delivery of equivalent doses (0.6 mg/kg lung dose) of aqueous versus dry powder 5AZA was conducted in the orthotopic LC model. Two adenocarcinoma tumour lines, Calu6 and Calu3, one in situ carcinoma H358, and one squamous cell carcinoma, RH2 derived from LC patients were evaluated. Three weeks following engraftment of tumour lines (lungs contain numerous tumours 1–5 mm [15]), rats (*n* = 15–20/group, see Methods) were exposed to air (untreated) or treated four times weekly for 4 weeks (same schedule as previous studies^23,24^) and then sacrificed to assess tumour burden. The dry powder was significantly better than the aqueous 5AZA with a 70–80% compared to 50% reduction in tumour burden for Calu6 and Calu3, respectively (Table 2). Treatment with either dry powder or aqueous 5AZA was equally effective in reducing the size of the H358 tumours ≥95% (Table 2). In contrast, the dry powder 5AZA was far superior in affecting growth of the aggressive squamous cell carcinoma RH2 (evident by the substantial tumour burden prior to initiation of treatment [Fig. 2a]) with a 74% reduction in burden compared to 33% for the aqueous formulation (Table 2).

**Table 2:** Superior efficacy of inhaled dry powder 5AZA on tumour burden in an orthotopic rat lung cancer model.

Treatment groups				
Cell line	Tumour type	Air	Aqueous	Dry powder
*Tumour Burden (gms)*				
Calu6	AdC	12.6 ± 4.3	6.2 ± 2.6^1^	2.4 ± 1.2^a,b^
Calu3	AdC	6.5 ± 2.1	3.1 ± 0.9^1^	1.9 ± 0.9^a,b^
H358	In Situ C	7.8 ± 3.6	0.4 ± 0.2^1^	0.3 ± 0.4^a^
RH2	SCC	14.1 ± 3.0	9.6 ± 2.1^1^	4.0 ± 1.9^a,b^

Mean ± SD from 13–19 rats/group.

*AdC* adenocarcinoma, *In Situ C* in situ carcinoma, *SCC* squamous cell carcinoma, *SCC* squamous cell carcinoma.

^a^*p* ≤ 0.001 comparing air control versus 5AZA aqueous or dry powder.

^b^*p* ≤ 0.003 comparing 5AZA aqueous versus dry powder.

Histopathology was performed on groups of five randomly selected rats. A second independent assessment of tumour burden was conducted by estimating the percentage of tumour tissue within tissue sections. In addition, mitotic activity and apoptosis/necrosis were assessed and scored accordingly. In general, the orthotopic xenografts were composed of coalesced nodules of neoplastic tissue growing in three dimensions that effaced much of the parenchyma and some of the peripheral bronchiolovascular tracts. Cytotoxicity, such as cytoplasmic vacuolisation and nuclear condensation or resulting effects of airway epithelial attenuation, hyperplasia or metaplasia were not evident in the airway epithelium or the alveoli of tumour-bearing and drug-exposed lungs.

The Calu6 adenocarcinomas were composed entirely of a pleomorphic cell population with a high mitotic rate (>50 mitoses in ten ×200 magnification fields). Cells were polygonal to cuboidal, arranged in solid nests, cords, and streams among a fine fibrovascular network (Fig. 2b). Calu3 adenocarcinomas showed a dimorphic pattern with expanses of relatively differentiated cells with low mitotic activity juxtaposed to regions of less differentiated, pleomorphic cells with greater mitotic activity. The differentiated cells were polygonal to cuboidal or columnar, sometimes regimented, and had abundant foamy to finely vesiculated cytoplasm consistent with secretory material (Fig. 2c). 5AZA therapy reduced the area of the lung occupied by tumour tissue in a manner consistent with the reductions seen for tumour burden assessed through weighing of the lungs at necropsy (Supplementary Table 1, Fig. 2d, e, f). For Calu3, the reduction in tumour mass largely affected the pleomorphic cell population. There was a slight decrease in mitotic activity in Calu6 and Calu3 tumours with the dry powder 5AZA (Supplementary Table 1).

More than 75% of the H358 tumour tissue was composed of pleomorphic cells that displayed morphological features similar to Calu6 with tumour cells arranged in nests and streams. Small contiguous foci of coagulative necrosis were scattered in the larger tumours along with infiltrates of neutrophils. Mitotic figures ranged from 25 to 50 per 10 high magnification fields and were reduced to 5–10 and <5 per 10 fields with aqueous versus dry powder 5AZA therapy (Supplementary Table 1). Lungs from untreated rats had 50–75% of tissue occupied by tumour and this was reduced to 5–25% and <5% with aqueous versus dry powder 5AZA therapy (Supplementary Table 1). Tumours remaining in the dry powder treatment group were often less than the diameter of a few alveoli and were often widely scattered in the parenchyma (Fig. 2g, h, i).

The RH2 tumours were multinodular and effaced much of the parenchyma along with pronounced infiltration of the perivascular and peribronchiolar interstitium. Tumours were pleomorphic, large regions contained pale cells with vacuolated cytoplasm, and centralised necrosis was often apparent (Fig. 2j). The number of mitotic figures was similar to that seen in H358 tumours; however, there was no significant treatment associated reduction (Supplementary Table 1). Tumours occupied >75% of lung tissue from RH2 untreated-treated rats. Consistent with burden assessment via total lung mass, 50–75% and ~25% of tissue mass was comprised of tumours in the aqueous and dry powder treatment groups, respectively (Supplementary Table 1). Interestingly, there did appear to be some increase in necrosis associated with either treatment (Fig. 2k, l).

### Treatment with 5AZA induces global demethylation of the epigenome

**Fig. 3:**
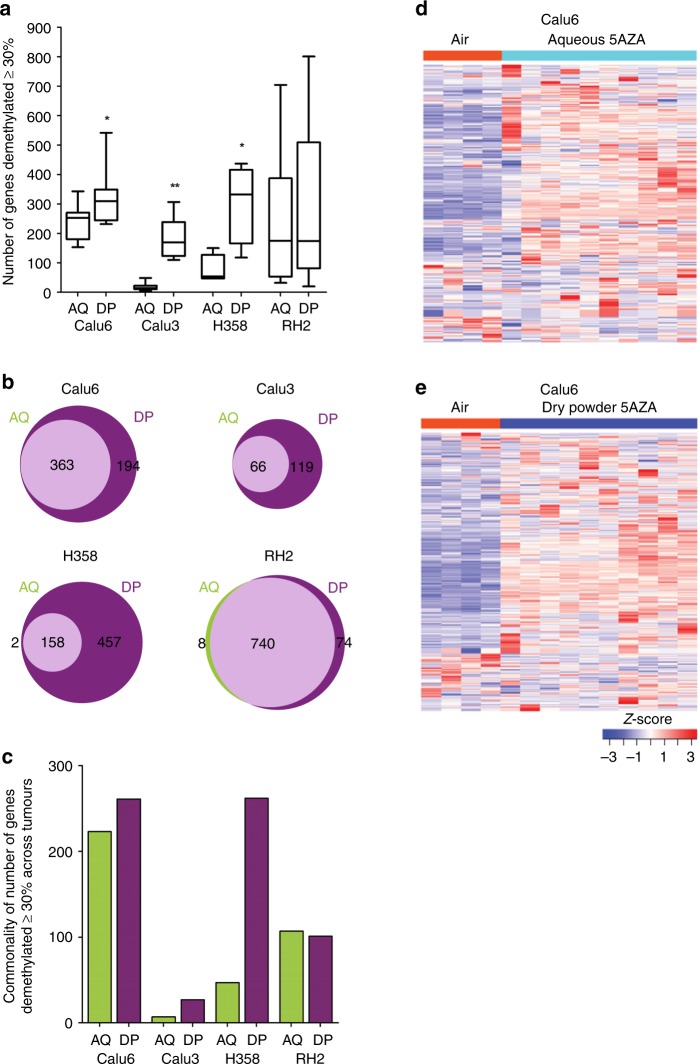
Increased global demethylation of the genome by dry powder 5AZA. Summary of Methylation EPIC Beadchip results using boxplots to depict total number of genes with ≥30% demethylation. The horizontal line within the boxes reflects the median and the whiskers indicate the range (min to max) and show increased number of genes methylated for Calu6 (*n* = 10), Calu3 (*n* = 10), H358 (*n* = 4) and RH2 (*n* = 10) tumours exposed to dry powder versus aqueous 5AZA (**a**). Venn diagrams show the common genes demethylated (middle) and the unique genes between therapies and across Calu6, Calu3, H358 and RH2 tumours (**b**). Commonality for number genes demethylated in ≥75% treated tumours by exposure and cell line is depicted by the bars (**c**). Expression level of genes demethylated in one or more aqueous or dry powder treated Calu6 tumours is displayed in the four air untreated rats and the effect of aqueous (**d**) and dry powder treatment (**e**) on gene re-expression is depicted across the 10 tumours in the heatmap. AQ, aqueous; DP, dry powder. Students *t*-test *p < 0.05 or **p < 0.01 compared to aqueous 5AZA.

The Illumina Infinium Methylation EPIC Beadchip was used to compare the effectiveness of aqueous vs dry powder formulation of 5AZA for inducing epigenome-wide demethylation. Four untreated tumours (1 from each rat), 10 tumours each from dry powder and aqueous exposed rats (either three or two tumours per lung to equal a total of 10) for Calu6, Calu3 and RH2 were studied. Due to the near curative effect of treatment for H358 only four discernible tumours from each treatment were available to support methylation and expression studies. After excluding genes methylated in normal lung tissue as described under Methods, the methylation state of 20,716 gene promoters defined by 200 bp 5’ of the transcriptional start site and extending through the first exon was determined in the untreated tumours. Genes were defined as methylated when the average β-value for probes interrogated within the promoter region was ≥0.45. The number of methylated genes in Calu6, Calu3, H358 and RH2 untreated tumours was 895, 558, 1081 and 849. Demethylation was defined as a reduction in β-value of ≥30% across an individual gene promoter. There was a significant increase in the number of demethylated genes in Calu6, Calu3 and H358 tumours exposed to dry powder versus aqueous 5AZA (Fig. 3a). The range for gene demethylation in RH2 was large across either treatment, extending from 25–800 genes, an effect reflecting the greater variation in response to treatment regarding reduction in tumour burden (Fig. 1, 3a). Venn diagrams of genes demethylated in at least one tumour per group capture both the commonality for effectiveness of the inhaled therapy route and the added effect of the dry powder formulation (Fig. 3b). For example, there were 363 genes demethylated across exposures in Calu6 tumours, zero unique genes associated with aqueous therapy, but an additional 194 genes demethylated by the dry powder formulation. These differences are further exemplified by increased commonality across tumours for demethylation for three of the four tumour types treated with dry powder vs. aqueous 5AZA (Fig. 3c).

### Superiority of the dry powder 5AZA formulation for transcriptional reprogramming of the genome

**Fig. 4:**
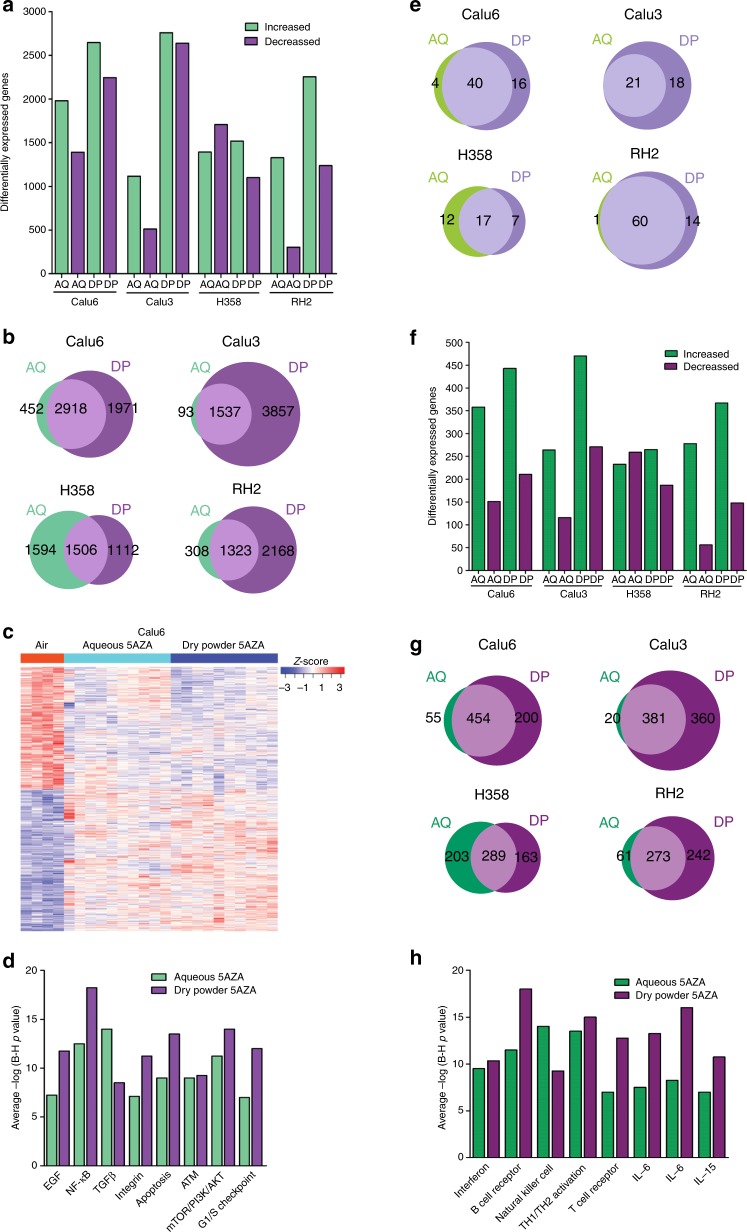
Superiority of the dry powder 5AZA formulation for transcriptional reprogramming the genome. The number of genes with increased or decreased expression (FDR < 0.01) across tumours from each treatment group is depicted by the bar graphs (**a**). Venn diagrams show the common differentially expressed genes (middle) and the unique genes between therapies across Calu6, Calu3, H358, and RH2 tumours (**b**). Heatmap of significantly differentially expressed genes comparing untreated to aqueous and dry powder 5AZA treatment in Calu6 tumours. Greater uniformity was seen across dry powder-treated tumours for increased and decreased expression compared to untreated tumours, while more heterogeneity was observed across aqueous-treated tumours (**c**). Average p-values across Calu6, Calu3, H358 and RH2 tumours show enrichment for genes residing in cancer signalling pathways by treatment (**d**). Venn diagrams show the common differentially expressed cancer testis antigens (middle) and the unique genes between therapies across Calu6, Calu3, H358, and RH2 tumours (**e**). The number of immunoregulatory genes with increased or decreased expression (FDR < 0.05) across tumours from each treatment group is depicted by the bar graphs (**f**). Venn diagrams show the common differentially expressed immunoregulatory genes (middle) and the unique genes between therapies across Calu6, Calu3, H358, and RH2 tumours (**g**). Average p-values across Calu6, Calu3, H358 and RH2 tumours show enrichment for genes residing in immune response pathways by treatment (**h**).

RNA-seq of untreated and treated tumours was used to evaluate the effect of epigenetic therapy on genome-wide gene expression profiles. More extensive differential expression across Calu6, Calu3 and RH2 was seen with the dry powder aerosol (Fig. 4a). Overall, the number of genes with increased and decreased expression (FDR ≤ 0.01) was 1117–2000 and 304–1708 for 5AZA aqueous-treated tumours and 1518–2757 and 1100–2637 for dry powder-treated tumours (Fig. 4a). Venn diagrams depict the large difference in unique differentially expressed genes (40–72% of total genes) seen with the dry powder treatment for all tumours except H358 (Fig. 4b). Heat maps best depict the distinct differential expression profiles seen across individual tumours exposed to aqueous versus dry powder 5AZA. For example, with Calu6 there was considerable uniformity across dry powder-treated tumours for increased and decreased expression compared to untreated tumours, while heterogeneity for expression changes was seen across aqueous-treated tumours (Fig. 4c). These differences in expression patterns were largely recapitulated for Calu3 and RH2 and to a lesser extent for H358 (Supplementary Fig. 1a–c). The relationship between gene demethylation and re-expression was also assessed using heat maps to visualise the change in expression on an individual tumour level for the demethylated genes and their expression in untreated versus treated tumours. Similar patterns of re-expression were seen in dry powder and aqueous aerosols across the different tumours and cell lines (Fig. 3d, e; Supplementary Fig. 2a–f). The heterogeneity for change in expression is consistent with the fact that most genes were not demethylated in all tumours from a cell line. A gene enrichment approach focused on cancer signalling was used for Ingenuity pathway analysis. Average p-values across Calu6, Calu3, H358 and RH2 tumours show enrichment for genes residing in the EGF, NF-κβ, TGF-β, mTOR/PI3K/AKT, integrin, ATM and G_1_/S checkpoint signalling pathways that was generally greater in tumours exposed to dry powder than aqueous 5AZA (Fig. 4d).

Epigenetic alterations during tumorigenesis play a key role in suppression of immune recognition and surveillance through modulating expression of tumour associated antigens that include the cancer testis antigens (CTAs) and a large number of genes involved in antigen processing and presentation.^33,34^ Therefore, we evaluated the effect of epigenetic therapy on CTAs and immunoregulatory genes involved in innate and adaptive immunity. The change in expression of 281 CTAs was assessed and the number of genes with increased expression (log2 fold change 0.35–10.1) ranged from 21–74 with the greatest effect seen in RH2-treated tumours. 5AZA therapy induced a large selection of CTAs, including members of the MAGE, SSX, SPANX and PAGE families (complete list in Supplementary Table 2). The Venn diagram depicts the greater magnitude of effect with the dry powder for Calu6, Calu3 and RH2 tumours along with the commonality and differences in number of affected genes in tumours as function of exposure (Fig. 4e). A combined list of 2529 immunoregulatory genes annotated in the ImmPort and InateDB databases were evaluated for change in expression (FDR < 0.05). The largest effect was seen for Calu3 tumours treated with dry powder 5AZA with ~30% of these genes differentially expressed (Fig. 4f). The expression patterns across tumours and exposures mirrored that seen for the global transcriptome changes with more extensive differential expression seen with the dry powder exposure. More than half the genes were common between dry powder and aqueous exposures, while again more unique genes showed altered expression in response to the dry powder 5AZA with exception being H358 where uniqueness was comparable (Fig. 4g). Immune response regulatory signalling showed an overwhelming enrichment of genes with increased expression in pathways that included interferon, B cell receptor, natural killer cell, TH1/TH2 activation, T cell receptor, IL-6, IL-8 and IL-15 (Fig. 4h). With the exception of genes involved in natural killer cell signalling, all other pathways showed enrichment that was greater in tumours from rats treated with dry powder than aqueous 5AZA.

## Discussion

These studies have developed and characterised an inhaled stabilised dry powder formulation of 5AZA whose pharmacokinetic profile and effectiveness for reducing tumour burden could be a paradigm shift for epigenetic therapy in the treatment of NSCLC. The 70–95% effectiveness of dry powder 5AZA on tumour burden of NSCLCs far surpassed our initial studies with the injectable formulation where only a 32% reduction was seen for the one cell line studied, Calu6.^23^ Inhaled delivery of the dry powder 5AZA was superior over the inhaled aqueous formulation and over the systemic dose for providing drug to the circulation, to the liver and most impressively to the brain with a half-life of 4 h. The dry powder formulation has different physiochemical properties when compared to the solution formulation as evident by the stability of the 5AZA. This improved stability and likely effects on solubility and dissolution likely account for the difference in plasma PK between the aqueous solution nebuliser and inhaled dry powder formulations of 5AZA and the apparent lung derived improvement in anti-tumour efficacy by the dry powder. These findings support the dry powder formulation and delivery route for not only treating localised lung cancer, but also importantly metastatic disease and potentially other solid tumours harbouring extensive epigenetically dysregulated genomes such as breast and colon cancer.^35,36^ Inhaled 5AZA may also have utility in adjuvant therapy for resected NSCLC, an area in which there have been no effective drugs.^37^

The plasma AUC and half-life for 5AZA of the subcutaneous dose of 75 mg/m^2^ used to treat myelodysplasia was shown to be 1147 ± 458 h*ng/ml and 1 ± 0.4 h in a study of 16 patients.^30^ This plasma pharmacokinetic profile is similar to that seen following the intraperitoneal dose of 2 mg/kg in rat (Table 1). Thus, we replicated the human systemic dose to compare with our inhaled delivery of a dry powder and aqueous formulation of 5AZA with respect to plasma bioavailability. While lung dose is not available for humans, there is no reason that the low lung dose seen with the intraperitoneal dose in the rat model would not reflect delivery to the human lung and other tissues. This supposition is supported by Qui et al.^17^ who used mice and showed a high dose of 5AZA in the peripheral blood with trace amounts in the lung following IV treatment, while the opposite scenario was observed with inhaled aqueous 5AZA. An oral formulation of 5AZA currently being evaluated in clinical trials is using a limited dose of 300–400 mg due to gastrointestinal toxicity that achieves a plasma AUC that is 70% lower than the subcutaneously delivered drug.^18,19,38^ Thus, it is unlikely that a therapeutically effective oral dose would reach the lungs. The dry powder 5AZA offers considerable flexibility for dose and delivery with capsule-based dry powder inhalers that require only minutes for administration. The inspiratory pressures related to dry powder inhalation drug delivery are driven by the muscle strength and tone within the diaphragm and the intercostal muscles that are not impacted by the disease state (e.g., tumour location or respiratory deficits) of the patient.^39^ NSCLCs exhibit dual blood supply via the bronchial and pulmonary vasculature, which along with direct deposition should facilitate drug delivery to the tumours.^21^ Finally, while toxicity of DMSO has not been evaluated via inhalation, intravenous dosing in rats at 200 mg/kg (2000 times greater than our inhaled 5AZA dose) for a month showed no toxicity and we observed no lung toxicity.^40^ Therefore, the patient population would be able to use the clinical devices required to deliver the inhaled dry powder 5AZA formulation.

While we cannot rule out the contribution of 5AZA mediated tumour cytotoxicity contributing to the reduction of tumour growth, it is likely minimal. 5AZA cytotoxicity manifests as inhibition of DNA and RNA synthesis.^41^ If this was a major mechanism, then the large effect on cytosine DNA demethylation state, which requires replicative incorporation into genomic DNA, would not have been seen. In fact, the greater potency for the inhaled dry powder versus aqueous 5AZA is likely due to its increased exposure of tumour tissue (caused by the greater chemical and physical stability) of the inhaled dry powder 5AZA that resulted in the stronger pharmacodynamic effect on genome-wide demethylation and reprogramming of the transcriptome. This was evident based on the increased number of distinct genes demethylated across tumours exposed to the dry powder compared to the aqueous formulation, irrespective of the cell line. This same finding was observed with respect to differential expression with exception being H358 tumours, an outcome potentially influenced by the small number of tumours available for RNA-seq due to the almost curative effect of either treatment. The significant change in gene expression with an FDR ≤ 0.01 involving 2600–5394 stems likely from direct and indirect effects of depleting DNMT1 by the dry powder 5AZA.^42^ DNMT1 is not only critical for maintaining hypermethylation of CpGs within gene promoters, but also participates in transcriptional repression by its presence in chromatin remodelling complexes with the histone methyltransferases EZH2 and G9a whose methylation at histone H3 lysine 27 and lysine 9 reduce gene transcription.^43–45^ The subsequent re-expression of these genes, many of which reside in key signalling pathways and are transcriptional regulators in turn positively or negatively affect expression of their targets. These effects were clearly evident through strong enrichment for genes in pathways involved in cell growth, differentiation, cytokine production, transcription, and DNA damage response.

Epigenetic alterations are used by tumour cells to impair immunogenicity and immune recognition.^33,34^ Several preclinical studies support that epigenetic reprogramming enhances immune recognition and response against cancer cells and reverses immune evasion.^46–48^ Thus, the effectiveness of inhaled epigenetic therapy in reprogramming the genome may better augment response to immunotherapy than current trials using oral drugs.^19^ Our study showed a strong effect on expression of CTAs known to enhance tumour cell targeting and lysis by MAG-A3 and CTAG-1B epitope-specific T cells.^49^ Pathway analysis of differentially expressed immune regulatory genes also predicts for significant effects of epigenetic therapy on antigen processing and presentation based on activation of interferon and cytokine signalling, B and T cell receptors, TH1/TH2 and natural killer cells. The strong response of lung tumours to epigenetic therapy through effects on cancer signalling pathways, along with activation of immune regulatory pathways and improved systemic delivery to extrapulmonary tissues that include brain, strongly support the premise that combining inhaled dry powder 5AZA with immunotherapy could shift the pendulum toward more effective management of metastatic disease.

## Supplementary information

Supplemental Material

## Data Availability

The authors declare that all data supporting the findings of this study are available within the article and its Supplementary Information files or are available from the corresponding author upon reasonable request.
