# Supplementary material for: 5-Azacytidine inhaled dry powder formulation profoundly improves pharmacokinetics and efficacy for lung cancer therapy through genome reprogramming
Source: Br J Cancer. 2020 Feb 27;122(8):1194–204. doi: 10.1038/s41416-020-0765-2 (PMC7156464; doi:10.1038/s41416-020-0765-2)
Supplement: Supplementary file 1 — Supplemental Material [file 41416_2020_765_MOESM1_ESM.docx]

**Supplemental Methods**

**Manufacturing and *in vitro* characterization of spray-dried powder of 5AZA**

5AZA (Toronto Research Chemicals, Inc. Toronto, Ontario, Canada) was dissolved at 5 mg/ml in DMSO (10% solvent mass) and H_2_O. The excipients trehalose and L-leucine at a ratio of 80/20 in H_2_O were added for stability and particle formation just prior to atomization. The solution was spray-dried using a two-fluid atomization nozzle with a small-scale custom spray dryer (BLD-35). Conditions were set to 500 g/min drying gas flow rate, 8 g/min liquid feed rate, 60 psi atomization pressure, 145°C inlet and 70°C outlet temperature. The analytical tests conducted to determine the physical, chemical, and aerosol characteristics of the dry powder formulation included; water content by Karl Fischer, particle size distribution (PSD) by Malvern Mastersizer wet-method, particle morphology by Scanning Electron Microscope (SEM), physical state by X-ray powder diffraction (XRPD), thermal properties by modulated differential scanning calorimetry (mDSC), and Potency/Purity by RP-HPLC. The aerosol properties of the spray-dried 5AZA powder including the mean mass aerodynamic diameter (MMAD), fine particle fraction (FPF), emitted faction (EF), geometric standard deviation (GSD) and powder distribution size plots were assessed using a RS01 clinical device (RS01, Plastiape, 4kPa) with a Next Generation Impactor (NGI model 170, MSP Corp.) operated at 100 L/min, for 2.4 seconds, and data from replicates were analyzed using Copley CITDAS Version 3.10. There was virtually no degradation of the 5AZA during the manufacturing process and purity of 92.4 ± 0.5% as assessed by reverse phase HPLC.

**Inhalation exposure, aerosol characterization, and pharmacokinetics**

All animal procedures were conducted under protocols approved by the Institutional Animal Care and Use Committee at Lovelace Respiratory Research Institute, which is accredited by the Association for Assessments and Accreditation of Laboratory Animal Care International. Rodents were housed in nose-only tubes during the exposures. Chamber oxygen content was collected directly from an enclosed nose-only exposure port. Temperature readings were obtained from a monitor placed within the secondary containment that enclosed the exposure chamber. All flow rates and system pressures were monitored by a rotameter (flow) and magnahelic (pressure) enclosed in a control panel. Flow rates were point-calibrated (calibrated at a specific flow rate) with a flow meter.

The inhaled pulmonary deposited doses were chosen based on our first study with the aqueous 5AZA aerosol. [1] The logic for that study was based on aerosol delivery being more potent per unit dose. Our target pulmonary deposited dose selected for 5AZA was 75% less than the effective systemic dose of 2 mg/kg. The actual calculated pulmonary deposited dose of 5AZA was based on standard inhalation drug delivery methods. [2] Thus, the current study directly compared the dry powder formulation to the aqueous formulation and also evaluated a lower and higher dose for the dry powder. This resulted in selection of the 0.3, 0.6, and 0.9 mg/kg pulmonary deposited doses.

The aerosol of the solution nebulizer 5AZA formulation (5 mg/ml) was generated with three Pari LC Plus nebulizers (Pari Respiratory Equipment, Midlothian, VA). The Pari LC Plus nebulizers were operated with clean, regulated inlet air at a pressure of 20 psi, which resulted in an output of ~ 15 L/min. The inhaled dry powder 5AZA aerosol was generated by a rotating brush generator (RBG 1000, Palas, Germany) and aerosol concentration adjustments were made by modulating the piston feed rate. The 5AZA aerosols were directed through ~ 24 in. of a 1.58-cm diameter stainless steel tube into a 52-port flow-past exposure chamber. The aerosol concentration of 5AZA (for both aqueous and inhaled dry powder) was measured at the breathing zone via aerosol filter collection. The filters were extracted and analyzed via HPLC-UV to determine the 5AZA aerosol concentration which was used to calculate pulmonary dose via standard methods.^26^ All exposures were conducted for 90 minutes to ensure uniformity across the study groups. The concentration of 5AZA aqueous and dry powder was stable through the exposure period based on similar levels on the three collected filters. The stability of aqueous 5AZA over the exposure period is also supported by Qui et al. [3] who collected the 5AZA aqueous aerosol fog 60 minutes after their exposure. They then treated the human NSCLC cell line H226 with the aerosol fog versus a non-aerosolized solution and reported the same inhibitory effect on cytosine DNA methyltransferase 1 (DNMT1) activity. This indicates that the basic epigenetic activity of 5AZA was preserved after 1 hour of aerosolization. While our exposure was for 90 minutes, their results and our filter data are consistent with regard to supporting minimal degradation of the aqueous 5AZA over the exposure period.

Pulmonary deposited doses were calculated as described for rodent inhalation studies. [2] Particle size distribution at the breathing zone of the exposure system was measured with a low flow cascade impactor (InTox, Moriarty, NM). In addition, the aerosol samples (filter samples) were shown to be stable for at least 14 days and the extracts were shown to be stable for at least 24 hours. All sample analysis was performed within these time windows.

**Bioanalysis of 5AZA**

The LC-MS/MS method used to analyze plasma and homogenized tissues from this study differed from Reed et al. [1] That method when re-evaluated did not provide satisfactory consistent resolution of the 5AZA peak because of inconsistency in the shape of the peak and background levels. This was also observed when reviewing chromatograms from Reed et al. and resulted in over estimating the amount of 5AZA in the plasma compartment in that study. [1] The resolution of 5AZA in the lung homogenates was also not as sharp as it should have been. While the message of the Reed et al. paper was not impacted by the less than optimal assessment of PK in plasma and lung (IP dose still higher in plasma than inhaled and inhaled 5AZA still higher in lung than IP), we felt that it was critical to develop a robust, sensitive, and accurate method for quantitation in plasma and tissues to support this work and future preclinical development.

To support this program, we used ^15^N_5_-deoxyadenosine as an internal standard along with a low to high dose range of 5AZA added to normal plasma or tissue homogenates to develop the standard curve. All methods (plasma, liver, lung and brain) were characterized for standard bioanalytical acceptance criteria (±15% and ±20% at LLOQ and ULOQ) with matrix-based standards and quality controls prior to analysis of samples. The plasma assay used a protein precipitation preparation method using 0.1% formic acid in acetonitrile followed by dilution with ammonium acetate buffer. Tissue assays used a homogenization step (1-part tissue with 4 parts 1x PBS) prior to protein precipitation. Tissue homogenates were prepared by a protein precipitation extraction using 10% trichloroacetic acid in water, followed by dilution with phosphate buffer. Homogenization of tissues was performed just prior to analysis. Separation was performed with a Waters HSS T3 (100Å, 3.5 µm, 3 mm X 100 mm) using a gradient with mobile phase A consisting of 10 mM ammonium acetate and mobile phase B consisting of 80/20 methanol/acetonitrile. The molecular weight of 5-AZA is 244.2 Da and the precursor → product ion transition (m/z 113.0 → 86.0) was monitored on a Sciex API 5000 triple quadrupole mass spectrometer, operating in multiple reaction monitoring and positive electrospray ionization mode. The new M/Z transition is actually a more selective method starting with the fragment ion of 5AZA to generate the product ion transition for quantitation. Quantification was performed with multiple reaction monitoring using matrix-based standards, quality controls, and ^15^N_5_-Deoxyadenosine as the internal standard. Supplemental Figure 3 shows representative chromatograms for the lowest (A) and a midlevel (C) dose of 5AZA and the internal standard (B, D). The retention times for 5AZA and ^15^N_5_-deoxyadenosine are 2.67 ± 0.5 and 3.18 ± 0.5 minutes. The plasma assay range was 50−10,000 ng/mL and the tissue assays had a range of 50 to 5,000 ng/g. Samples that were above the upper limit of quantitation were diluted and re-run. Precision and accuracy for the plasma method ranged from ~ 89% to 110% accuracy for the standard and between 97 and 103% (~ 5% CV) for the QC’s. Precision and accuracy for the lung method ranged from 97 to 107% accuracy for the standards and between 90 and 102% (2 – 5% CV) for the QC’s. Precision and accuracy for the brain method ranged from 99 to 110% accuracy for the standards and between 92 and 108% (~ 8% CV) for the QC’s. Precision and accuracy for the liver method ranged from 93 to 113% accuracy for the standards and between 90 and 102% (~ 9% CV) for the QC’s.

**Statistical analyses**

Pharmacokinetic (PK) parameters were estimated for plasma, lung, liver, and brain using Phoenix WinNonlin version 6.2 software (Certara L.P.) using a non-compartmental (NCA) approach. Concentrations below limit of quantification were treated as missing for the analysis. PK parameters (T_max_, C_max_, AUC_last_) were calculated for each tissue/delivery group/dose group using NCA sparse data methodology, in which parameters were calculated based on the mean profile of all subjects in the data set, but the subject information is used to account for any correlations in data resulting from repeated sampling of individual animals. The mean concentration curve’s maximum value (C_max_) and area under the mean concentration curve from time zero to the time at which the last quantifiable concentration was observed (AUC_last_) for each tissue, was calculated using the linear trapezoidal method with linear/log interpolation. Standard error of the mean C_max_ was calculated as the sample standard error of the concentration values at T_max_, and standard error of the mean AUC was calculated as described in Nedelman and Jia, using a modification in Holder. [4, 5] The apparent terminal elimination phase of each concentration versus time curve was identified using at least the final three observed concentration values. The slope of the terminal elimination phase was determined using log regression with uniform weighting. The apparent terminal elimination half-life derived from the apparent terminal elimination phase was reported if it passed the reporting criteria: the coefficient of determination (R^2^) was greater than or equal to 0.9 and the extrapolation of the AUC to infinity was less than or equal to 30% of the total area. Linear mixed modeling in Phoenix WinNonlin tested the overall effect of the difference between delivery methods (by comparing the 0.6 mg/kg aerosol dry powder group versus the aqueous aerosol and systemic delivery route, and the difference between the different doses of inhalation dry powder 5AZA (0.3, 0.6, and 0.9 mg/kg) separately in each tissue. Fixed effect terms for time and delivery route (or dose if there was no difference in delivery route), and repeated animal were included to predict tissue concentration in the model.

The two-sample t-test and analysis of variance were used to compare tumor burden between the two treatment groups and the two groups with the air, respectively.

Due to the strong association between methylation of CpGs around the transcriptional start site (TSS) and gene silencing, our analytic strategy for methylation data focused on this region to assess the methylation status of 179,314 CpG oligonucleotide probes within 200 base pairs 5' of the TSS and extending through the first exon. Array iIdat files were imported into R (3.5.1) for preprocessing using the minfi package (Bioconductor 3.5). Raw signal intensities were normalized using noob background subtraction with dye-bias normalization. Probe qualities within arrays were assessed using the detection P-function and probes with p-values >0.01 in any sample were removed. Array data for each cell line were processed and analyzed separately. Average signal intensity between methylated and unmethylated probes was determined, and β-values from 0-1 (fully methylated) were calculated. Genes whose average β-values were ≥ 0.2 across the interrogated region in normal lung tissue were excluded from further analysis. Average β-values ≥ 0.45 across CpGs within gene promoters were scored as positive for methylation in untreated tumors and a reduction in β-value of ≥ 30% for a methylated gene in treated tumors was scored as demethylation. Analyses were conducted with SAS 9.4.

RNA sequencing data were analyzed using Illumina’s cloud-based genomics-computing environment. Sequencing reads were filtered and trimmed using Bowtie, aligned to a human reference genome (UCSC hg19) using STAR, and quantified using Salmon through Illumina’s RNA-Seq Alignment app v2.0. The count-based statistical method DESeq2 in Illumina’s RNA-Seq Differential Expression app v1.01 was used to identify differences (fold changes) between treated tumorss relative to controls. DeSeq2 uses the Wald test for significance testing and Benjamini-Hochberg procedure for multiple testing adjustment. Heatmaps were generated in R. The color gradation reflects the z-score ranking of rlog values from DESeq2 analyses.

Genes from pathways affected in cancer were compiled from Biocarta, mSigdb and IPA knowledge base and used to assess over-enrichment in significantly differentially expressed genes (FDR <0.01) in each group of treated tumors. Qiagen Ingenuity Pathway Analysis software was used to identify pathways statistically over-represented in the lists of differentially expressed genes.

**References**

1. Reed, M., Tellez, C., Grimes, M., Picchi, M., Chen, Y., March, T., et al. Aerosolized 5‑azacytidine suppresses tumor growth and reprograms the epigenome in an orthotopic lung cancer model. Br J Cancer **109**, 1775-1781 (2013).
2. Tepper, J., Kuehl, P., Cracknell, S., Nikula, K., Pei, L., Blanchard, J. Symposium summary: Breathe in, breath out, its easy: What you need to know about developing inhaled drugs. Int J Toxicol. **35**, 376-392 (2016).
3. Qiu, X., Liang, Y., Sellers, R., Perez-Soler, R., Zou, Y. Toxicity and pharmacokinetic studies of aerosolized clinical grade azacytidine. Clin Lung Cancer **3**, 214-222 (2015).
4. Nedelman, J., Jia, X. An extension of Satterthwaite's approximation applied to pharmacokinetics. J Biopharm Stat. **8**, 317-328 (1998).
5. Holder, D. Comments on Nedelman and Jia's extension of Satterthwaite's approximation applied to pharmacokinetics. J Biopharm Stat. **11**, 75-79 (2011).

**Supplemental Figure Legends**

**Supplemental Fig. 1** Heatmap of significantly differentially expressed genes comparing air to aqueous and dry powder 5AZA treatment in Calu3 (a), H358 (b), and RH2 (c) tumors.

**Supplemental Fig. 2**  Expression levels of genes demethylated in Calu3 (a, b), H358 (c, d), and RH2 (e, f) tumors. The expression level of genes demethylated in one or more treated tumors is displayed in the 4 air exposed rats and the effect of aqueous and dry powder treatment is depicted across the treated tumors in the heatmap.

**Supplemental** **Figure 3.** Representative chromatograms for the lowest (100 ng/ml [A]) and a midlevel (2000 ng/ml [C]) dose of 5AZA and the ^15^N_5_-deoxyadenosine internal standard (B, D). The retention times for 5AZA and ^15^N_5_-deoxyadenosine are 2.67 ± 0.5 and 3.18 ± 0.5 minutes.

**Supplemental Table 1. Effect of inhaled 5AZA on tumor area, mitosis, and apoptosis**

Cell Line Treatment Tumor Area Apoptosis Mitotic Activity

(% grade) (# Grade) (# Grade)

Calu6 Air 4.4 ± 0.6 2.6 ± 0.6 4.0 ± 0.0

Aq 5AZA 3.8 ± 0.5 2.2 ± 0.5 4.0 ± 0.0

DP 5AZA 1.8 ± 0.5** 1.8 ± 0.5 3.4 ± 0.6

Calu3 Air 3.4 ± 0.6 1.8 ± 0.5 1.6 ± 0.6

Aq 5AZA 3.0 ± 0.0 1.6 ± 0.6 1.6 ± 0.6

DP 5AZA 2.0 ± 0.0** 1.0 ± 0.0 1.0 ± 0.0

H358 Air 4.2 ± 0.5 2.0 ± 0.7 2.4 ± 0.6

Aq 5AZA 1.8 ± 0.5** 1.0 ± 0.0 1.4 ± 0.6*

DP 5AZA 1.0 ± 0.0** 0.8 ± 0.5 0.4 ± 0.6**

RH2 Air 4.8 ± 0.5 3.2 ± 0.5 2.0 ± 0.0

Aq 5AZA 4.0 ± 0.0* 4.0 ± 0.0* 1.8 ± 0.8

DP 5AZA 2.6 ± 0.6** 4.0 ± 0.0* 1.6 ± 0.6

Aq, aqueous; DP, dry powder; hpfs, high power magnification fields at 20X magnification.

Tumor area % grade: 0 = absent, 1 = <5%, 2 = 5-25%, 3 = >25-50%, 4 = >50-75%, 5 = >75%.

Apoptosis/necrosis grade: 0 = absent, 1 = minimal, 2 = mild, 3 = moderate, 4 = marked.

Mitotic activity grade: 1 = 1-5 figures, 2 = 6-25, 3 = 26-50, and 4 = >50 per 10 hpfs.

*p < 0.05, **p<0.01 for one-sided exact Wilcoxon p-values compared to air, mean ± SD, n = 5/group

| **Supplemental Table 2. Cancer testis antigens with increased expression following treatment with 5AZA** | | | | |
| --- | --- | --- | --- | --- |
|  |  |  |  |  |
| **Aqueous 5AZA** |  |  |  | **Dry Powder 5AZA** |
| **Gene** |  |  |  | **Gene** |
| ACTL8 |  |  |  | ACTL8 |
| ANKRD45 |  |  |  | ANKRD45 |
| ARMC3 |  |  |  | BRDT |
| BRDT |  |  |  | CABYR |
| CABYR |  |  |  | CCDC36 |
| CCDC36 |  |  |  | CEP290 |
| CEP55 |  |  |  | CEP55 |
| COX6B2 |  |  |  | COX6B2 |
| CSAG1 |  |  |  | CSAG1 |
| CT45A1 |  |  |  | CT45A1 |
| CT45A3 |  |  |  | CT45A3 |
| CT45A6 |  |  |  | CT47B1 |
| CTAG1A |  |  |  | CTAG1A |
| CTAG1B |  |  |  | CTAG1B |
| CTAG2 |  |  |  | CTAG2 |
| CTCFL |  |  |  | CTCFL |
| DDX43 |  |  |  | CTNNA2 |
| DPPA2 |  |  |  | DDX43 |
| DSCR8 |  |  |  | DDX53 |
| FAM133A |  |  |  | DPPA2 |
| FMR1NB |  |  |  | DSCR8 |
| FTHL17 |  |  |  | ELOVL4 |
| GAGE1 |  |  |  | FAM133A |
| GAGE12J |  |  |  | FMR1NB |
| GAGE2A |  |  |  | FTHL17 |
| GAGE8 |  |  |  | GAGE1 |
| GPAT2 |  |  |  | GAGE12J |
| HORMAD1 |  |  |  | GAGE2A |
| IL13RA2 |  |  |  | GAGE8 |
| LOC440934 |  |  |  | GPAT2 |
| LUZP4 |  |  |  | HORMAD1 |
| LY6K |  |  |  | HSPB9 |
| MAEL |  |  |  | IL13RA2 |
| MAGEA1 |  |  |  | KIAA0100 |
| MAGEA10 |  |  |  | LOC440934 |
| MAGEA11 |  |  |  | LUZP4 |
| MAGEA12 |  |  |  | LY6K |
| MAGEA2 |  |  |  | MAEL |
| MAGEA2B |  |  |  | MAGEA1 |
| MAGEA3 |  |  |  | MAGEA10 |
| MAGEA4 |  |  |  | MAGEA11 |
| MAGEA6 |  |  |  | MAGEA12 |
| MAGEA8 |  |  |  | MAGEA2 |
| MAGEA9 |  |  |  | MAGEA2B |
| MAGEB1 |  |  |  | MAGEA3 |
| MAGEB2 |  |  |  | MAGEA4 |
| MAGEB6 |  |  |  | MAGEA6 |
| MAGEC1 |  |  |  | MAGEA8 |
| MAGEC2 |  |  |  | MAGEA9 |
| MAGEC3 |  |  |  | MAGEB1 |
| NXF2 |  |  |  | MAGEB2 |
| ODF2 |  |  |  | MAGEB6 |
| OIP5 |  |  |  | MAGEC1 |
| PAGE1 |  |  |  | MAGEC2 |
| PAGE2 |  |  |  | MAGEC3 |
| PAGE2B |  |  |  | MORC1 |
| PAGE5 |  |  |  | NR6A1 |
| PASD1 |  |  |  | NXF2 |
| PIWIL2 |  |  |  | NXF2B |
| PRAME |  |  |  | ODF2 |
| RBM46 |  |  |  | PAGE1 |
| SAGE1 |  |  |  | PAGE2 |
| SPA17 |  |  |  | PAGE2B |
| SPAG1 |  |  |  | PAGE5 |
| SPAG4 |  |  |  | PASD1 |
| SPAG6 |  |  |  | PIWIL2 |
| SPANXA1 |  |  |  | PLAC1 |
| SPANXB1 |  |  |  | POTEE |
| SPANXC |  |  |  | PRAME |
| SPANXD |  |  |  | RBM46 |
| SPANXN4 |  |  |  | SAGE1 |
| SPEF2 |  |  |  | SPA17 |
| SSX1 |  |  |  | SPAG1 |
| SSX3 |  |  |  | SPAG17 |
| SSX4 |  |  |  | SPAG4 |
| SSX5 |  |  |  | SPAG6 |
| SYCE1 |  |  |  | SPAG9 |
| TAF7L |  |  |  | SPANXA1 |
| TDRD1 |  |  |  | SPANXB1 |
| TEX101 |  |  |  | SPANXC |
| TEX14 |  |  |  | SPANXD |
| TEX15 |  |  |  | SPANXN4 |
| TFDP3 |  |  |  | SPEF2 |
| TMEFF1 |  |  |  | SSX1 |
| TMEFF2 |  |  |  | SSX3 |
| XAGE1B |  |  |  | SSX4 |
| XAGE3 |  |  |  | SSX5 |
|  |  |  |  | SYCE1 |
|  |  |  |  | TAF7L |
|  |  |  |  | TDRD1 |
|  |  |  |  | TEKT5 |
|  |  |  |  | TEX101 |
|  |  |  |  | TEX14 |
|  |  |  |  | TEX15 |
|  |  |  |  | TFDP3 |
|  |  |  |  | THEG |
|  |  |  |  | TMEFF2 |
|  |  |  |  | TMEM108 |
|  |  |  |  | XAGE1B |
|  |  |  |  | XAGE3 |
|  |  |  |  | ZNF165 |

| **a**  **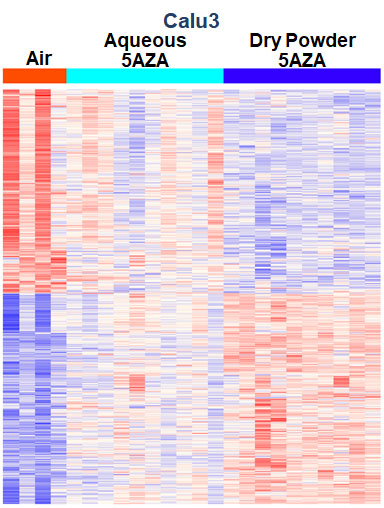** |  | **b**  **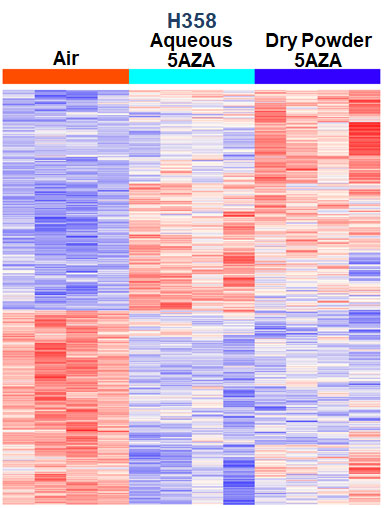** |
| --- | --- | --- |
| **c**  **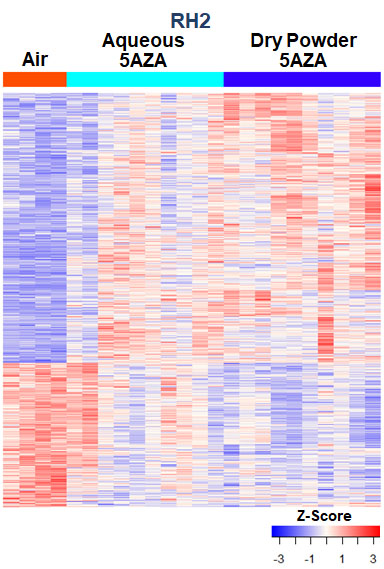** |  |  |

Supplemental Figure 1a, b, and c

| **a**  **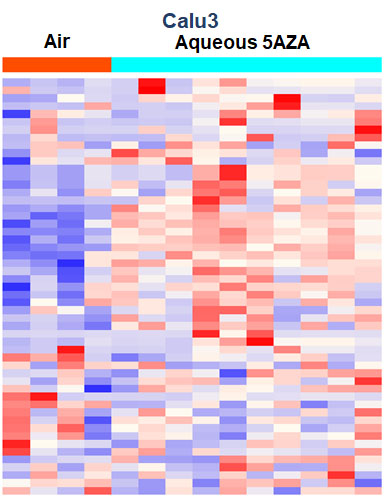** |  | **b**  **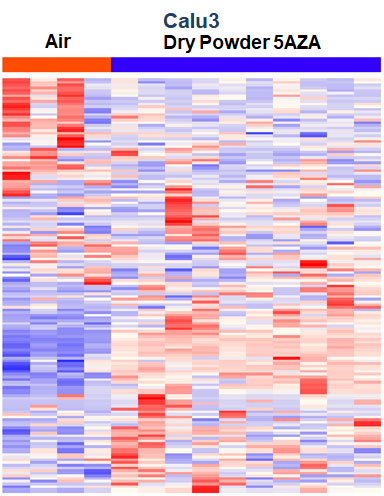** |
| --- | --- | --- |
| **c**  **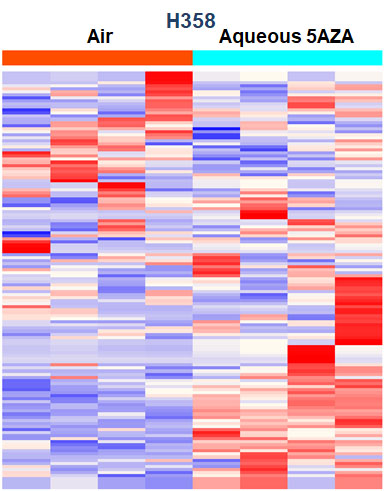** |  | **d**  **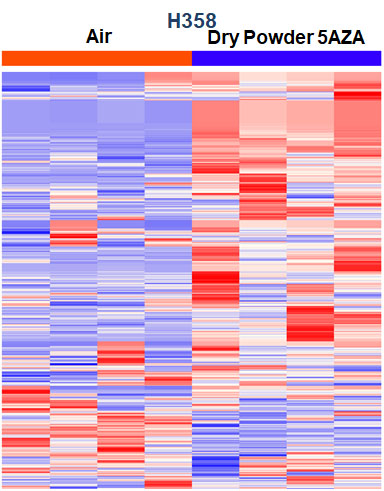** |
| **e**  **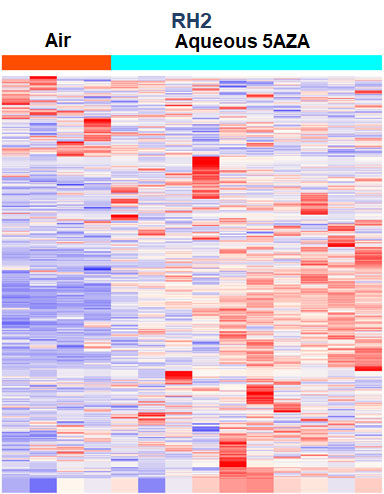** |  | **f**  **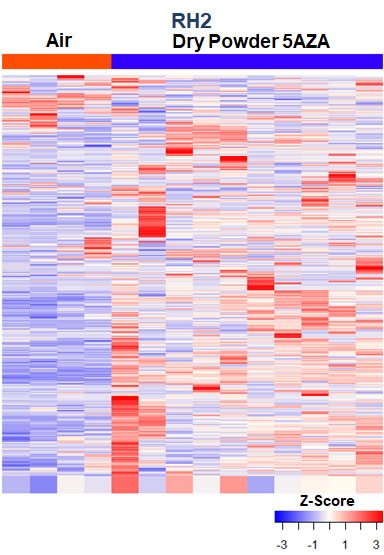** |

Supplemental Figure 2 a, b, c, d, e, and f

A. B.


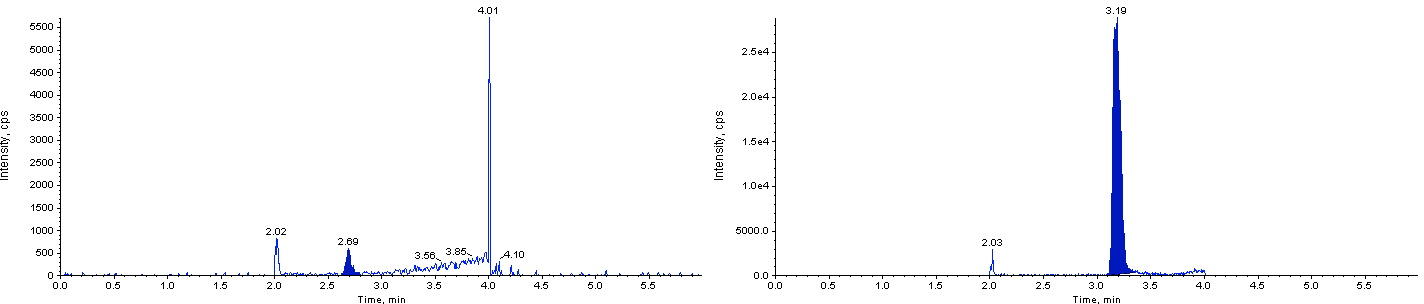


C. D.


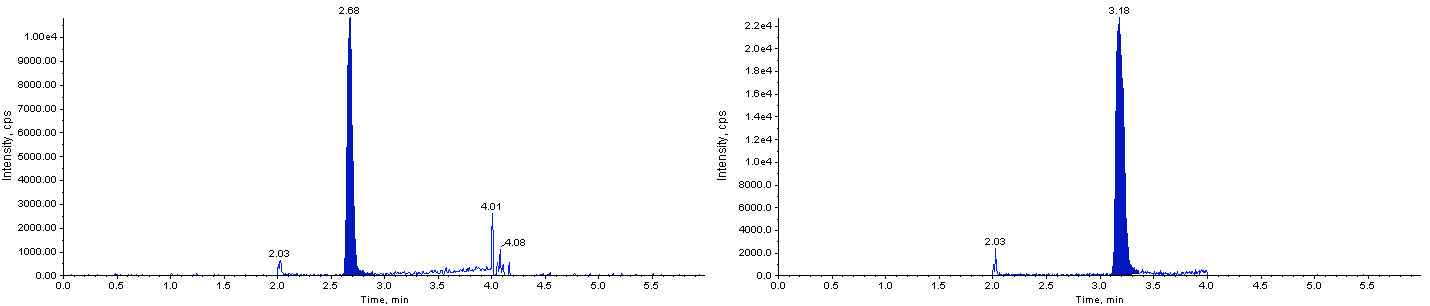


Supplemental Figure 3.
